# Supplementary material for: A new mechanism for a familiar mutation – bovine DGAT1 K232A modulates gene expression through multi-junction exon splice enhancement
Source: BMC Genomics. 2020 Aug 26;21:591. doi: 10.1186/s12864-020-07004-z (PMC7449055; doi:10.1186/s12864-020-07004-z)
Supplement: Supplementary file 1 — Additional file 1 Supplementary Figure 1. Schematic of the 5′ end of DGAT1 exon 8 with ESE motifs overlapping the K232A amino acid substitution. Supplementary Table 1. Mammary DGAT1 expression association statistics for top WGS-derived variants. Supplementary Table 2. Mammary DGAT1 expression association statistics for top sequence variants conditioned on DGAT1 K232A. Supplementary Table 3. Primer sequences and assay design for RT-qPCR of DGAT1 introns 3, 5, 7, and 13. [file 12864_2020_7004_MOESM1_ESM.docx]

**Supplementary Figure 1. Schematic of the 5′ end of *DGAT1* exon 8 with ESE motifs overlapping the K232A amino acid substitution.**

The AA>GC MNP responsible for the K232A substitution is underlined in the *DGAT1* K allele.


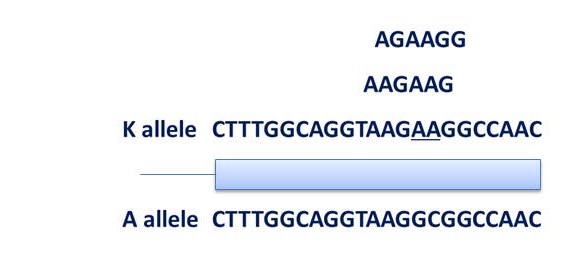


**Supplementary Table 1**. **Mammary *DGAT1* expression association statistics for top WGS-derived variants**

The positions of these SNP variants are indicated, with parameter estimates shown with standard errors in units of VST-transformed RNAseq read counts. The genetic and phenotypic variance explained by each SNP, along with parameter-adjusted means for each of the three genotypes classes is indicated. The linkage disequilibrium R^2^ values for each SNP relative to the *DGAT1* K232A variant is shown, with the P-values indicated in the right most column.

|  |  |  | **Adjusted means** | | |  |  |  |  |
| --- | --- | --- | --- | --- | --- | --- | --- | --- | --- |
| Variant | **Chr14 pos** | **Parameter Est** | **Geno 0** | **Geno 1** | **Geno 2** | **Pheno var** | **Geno var** | **R^2^ with K232A** | **P-value** |
| rs209328075 | 1730455 | 0.1927(±0.0161) | 9.251 | 9.446 | 9.642 | 31.32 | 99.99 | 0.881 | 2.31×10^−28^ |
| rs209929366 | 1747132 | 0.1927(±0.0161) | 9.251 | 9.446 | 9.642 | 31.32 | 99.99 | 0.881 | 2.31×10^−28^ |
| rs208091850^*^ | 1722033 | 0.1961(±0.0164) | 9.244 | 9.440 | 9.637 | 31.23 | 99.99 | 0.922 | 1.29×10^−27^ |
| rs208417762^^^ | 1756075 | 0.1969(±0.0166) | 9.240 | 9.437 | 9.634 | 31.33 | 99.99 | 0.968 | 2.38×10^−27^ |
| rs135458711^+^ | 1724688 | 0.1906(±0.0167) | 9.250 | 9.440 | 9.631 | 29.46 | 99.99 | 0.952 | 1.10×10^−25^ |
| K232A^&^ | 1802265 | 0.1919(±0.0169) | 9.244 | 9.436 | 9.628 | 29.71 | 99.99 | 1 | 1.59×10^−25^ |

^*^31, ^^^27, ^+^7 and ^&^20 additional genetic variants, respectively had the same association signal for *DGAT1*. These variants were statistically indistinguishable from each other and are not included in this table in the interest of size.

**Supplementary Table 2.** **Mammary *DGAT1* expression association statistics for top sequence variants conditioned on *DGAT1* K232A**

The positions of these SNP variants are indicated, with parameter estimates shown with standard errors in units of VST-transformed RNAseq read counts. The genetic and phenotypic variance explained by each SNP, along with parameter-adjusted means for each of the three genotypes classes is indicated. The linkage disequilibrium R^2^ values for each SNP relative to the *DGAT1* K232A variant is shown, with the P-values indicated in the right most column.

|  |  |  | **Adjusted means** | | |  |  |  |  |
| --- | --- | --- | --- | --- | --- | --- | --- | --- | --- |
| Variant | **Chr14 pos** | **Parameter Est** | **Geno 0** | **Geno 1** | **Geno 2** | **Pheno var** | **Geno var** | **R^2^ with K232A** | **P-value** |
| rs472613236* | 1721117 | 0.1013(±0.0242) | 9.361 | 9.463 | 9.564 | 10.32 | 99.99 | 0.548 | 3.58x10^−5^ |
| rs383105805^ | 1428907 | 0.1002(±0.0240) | 9.363 | 9.463 | 9.564 | 10.13 | 99.99 | 0.539 | 3.88x10^−5^ |
| rs109448144 | 1704351 | −0.1004(±0.0240) | 9.564 | 9.464 | 9.364 | 10.06 | 99.99 | 0.544 | 4.14x10^−5^ |
| rs137587412^&^ | 1438890 | 0.0975(±0.0243) | 9.366 | 9.463 | 9.561 | 9.62 | 99.99 | 0.546 | 7.08x10^−5^ |
| rs445906781 | 1723278 | 0.1165(±0.0295) | 9.424 | 9.540 | 9.657 | 4.95 | 99.99 | 0.090 | 9.71x10^−5^ |
| rs476272800 | 1754446 | 0.1165(±0.0295) | 9.424 | 9.540 | 9.657 | 4.88 | 99.99 | 0.853 | 9.71x10^−5^ |

^*^39, ^^^9 and ^&^5 additional genetic variants, respectively had the same association signal for *DGAT1*. These variants were statistically indistinguishable from each other and are not included in this table in the interest of size.

**Supplementary Table 3: Primer sequences and assay design for RT-qPCR of *DGAT1* introns 3, 5, 7, and 13.**

| ***DGAT1* Intron** | **Probe** | **Primers** |  |
| --- | --- | --- | --- |
| 3 | 9 | F1 | ACTACCGTGGCATCCTGAAT |
|  |  | F2 | CAGTTCTGACAGTGGCTTCAG |
|  |  | R | CACCAGGATGCCATACTTGAT |
| 5 | 66 | F1 | CGTTCCAGGTGGAGAAGC |
|  |  | F2 | GTGGGAGCTCTGACGGAG |
|  |  | R | GAATGGTGGCCAGGTTGA |
| 7 | 57 | F | TCAAGCTGTTCTCCTACCGG |
|  |  | R1 | CGAGGCAGCCCTCACCAG |
|  |  | R2 | CTTACCTGCCAAAGCAGC |
| 13 | 71 | F | CACTTCTACAAGCCCATGCTC |
|  |  | R1 | CTTCACCGGCATGATGGC |
|  |  | R2 | CACCAGGTACTCGTGGAAGAA |
| **Control Genes** |  |  |  |
| **EIF3K** | 1 | F | AAGTTGCTCAAGGGGATCG |
|  |  | R | TTGGCCTGTGTCTCCACATA |
| **GFP** | 5 | F | CGACGGCGGCTACTACAG |
|  |  | R | GTGGATGGCGCTCTTGAA |
